# The Role of Non-Coding RNAs in Uveal Melanoma

**DOI:** 10.3390/cancers12102944

**Published:** 2020-10-12

**Authors:** Manuel Bande, Daniel Fernandez-Diaz, Beatriz Fernandez-Marta, Cristina Rodriguez-Vidal, Nerea Lago-Baameiro, Paula Silva-Rodríguez, Laura Paniagua, María José Blanco-Teijeiro, María Pardo, Antonio Piñeiro

**Affiliations:** 1 Department of Ophthalmology, University Hospital of Santiago de Compostela, Ramon Baltar S/N, 15706 Santiago de Compostela, Spain; daniel.fernandez.diaz@rai.usc.es (D.F.-D.); beatriz.fernandez.marta@rai.usc.es (B.F.-M.); mariajose.blanco@usc.es (M.J.B.-T.); antonio.pineiro@usc.es (A.P.); 2 Tumores Intraoculares en el Adulto, Instituto de Investigación Sanitaria de Santiago (IDIS), 15706 Santiago de Compostela, Spain; paula.silva.rodriguez@rai.usc.es (P.S.-R.); maria.pardo.perez@sergas.es (M.P.); 3 Department of Ophthalmology, University Hospital of Cruces, Cruces Plaza, S/N, 48903 Barakaldo, Vizcaya, Spain; cristina.rodriguez.vidal@rai.usc.es; 4 Grupo Obesidómica, Instituto de Investigación Sanitaria de Santiago (IDIS), 15706 Santiago de Compostela, Spain; nerea.lago@rai.usc.es; 5 Fundación Pública Galega de Medicina Xenómica, Clinical University Hospital, SERGAS, 15706 Santiago de Compostela, Spain; 6 Department of Ophthalmology, University Hospital of Coruña, Praza Parrote, S/N, 15006 La Coruña, Spain; laura.paniagua@rai.usc.es

**Keywords:** uveal melanoma, ncRNAs, miRNA, lncRNAs, review

## Abstract

**Simple Summary:**

The development of uveal melanoma is a multifactorial and multi-step process, in which abnormal gene expression plays a key role. Recently, several studies have highlighted the role of non-coding RNAs in the progression of uveal melanoma by affecting different signaling pathways. As important agents in the regulation of genes, non-coding RNAs have enormous potential to open up therapeutic pathways, predict response to treatment, and anticipate patient outcome for uveal melanoma. This review aims to provide a comprehensive view of what we know about ncRNAs in uveal melanoma currently.

**Abstract:**

Uveal melanoma (UM) is the most common primary intraocular tumor in adulthood. Approximately 50% of patients develop metastatic disease, which typically affects the liver and is usually fatal within one year. This type of cancer is heterogeneous in nature and is divided into two broad groups of tumors according to their susceptibility to develop metastasis. In the last decade, chromosomal abnormalities and the aberrant expression of several signaling pathways and oncogenes in uveal melanomas have been described. Recently, importance has been given to the association of the mentioned deregulation with the expression of non-coding RNAs (ncRNAs). Here, we review the different classes of ncRNAs—such as long non-coding RNAs (lncRNAs) and microRNAs (miRNAs)—and their contribution to the development of UM. Special attention is given to miRNAs and their regulatory role in physiopathology and their potential as biomarkers. As important agents in gene regulation, ncRNAs have a huge potential for opening up therapeutic pathways, predicting response to treatment, and anticipating patient outcome for UM.

## 1. Introduction

**Figure 1:**
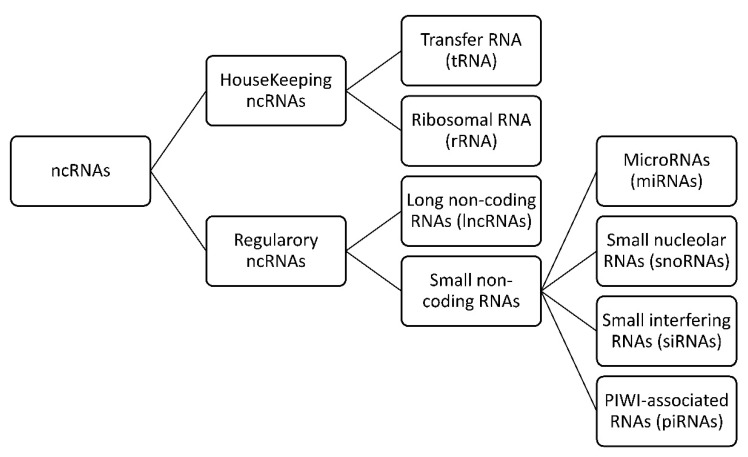
ncRNA categories. RNAs are divided into two major classes: messenger RNA (mRNA) and non-coding RNA (ncRNA).

The latest research on the human genome indicates that only 2% of human DNA is encodes proteins [1]. However, it is known that over 80% of the human genome contains elements linked to biochemical functions [2]. What was formerly considered junk DNA has more recently been demonstrated to be ncRNAs that perform multiple biologic processes and whose deregulation may be associated with multiple diseases, including cancer [3,4]. In addition to the well-known types of ncRNA, such as tRNA and rRNA that are involved in protein synthesis, ncRNA also comprises many new transcripts that have been identified in the last decade. They are divided into two groups according to their size: small ncRNA and long ncRNA. The size of small ncRNAs is usually less than 200 nucleotides and includes miRNA, PIWI-interacting RNA (piRNA), small nucleolar RNA (snoRNA), and promoter-associated small RNA (PASR) [5], as summarized in Figure 1.

Uveal melanoma (UM) is the most common primary intraocular malignancy in adults. At present, using any of the conservative treatment options or combinations results in a degree of local control of greater than 90% after 5 years. Despite this success, metastatic disease appears in more than 50% of patients after 15 years of local treatment. This poor prognosis is associated with clinical and molecular factors of primary UM, such as tumor height, presence of monosomy 3, and chromosome 8 gain, while 6p gain provides a protective effect [6]. Genetic prognostic markers have been found that can be used to identify patients at risk of developing metastatic disease. Gene expression profiles (GEPs) are used to classify UMs for disease-specific mortality risk, with class 1A being very low risk (2% risk at 5 years), class 1B being low risk (21% risk at 5 years), and class 2 being high risk (72% at 5 years) [7]. Likewise, tumor-specific mutations have been found in the genes *GNAQ, GNA11, EIF1AX, SF3B1*, and *BAP1* [8,9].

In recent years, many studies have shown that ncRNAs play a key role in multiple biological processes in UM, such as tumorigenesis, proliferation, and metastasis. Some ncRNAs have also been identified as biomarkers for the clinical diagnosis and prognosis of UM. In this review, we summarize the role and mechanisms of different ncRNAs in the physiopathology of UM, including those that may have a clinical application in the diagnosis and prognosis of this type of cancer.

## 2. MicroRNAs in UM

Of all the types of ncRNAs, miRNAs are the best-studied and understood within the context of UM initiation and progression. MicroRNAs—RNA strands of about 17–22 nucleotides—are estimated able to regulate 60% of all protein-encoding genes, and a single miRNA can regulate up to 400 different mRNAs [10]. They regulate the expression of genes both at the transcriptional and post-transcriptional level and are implicated in several physiological and pathological processes in which they exert their regulatory effects through binding of the 3′-UTRs of target mRNAs [11,12].

### 2.1. Dysregulated Expression of MicroRNAs

**Figure 2:**
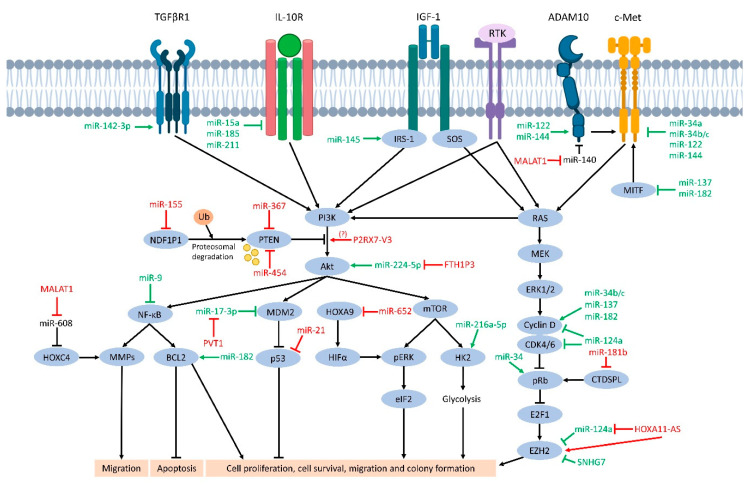
The impact of ncRNAs-mediated PI3K/AKT and MAPK/ERK pathway in uveal melanoma (UM). The ncRNAs that have a suppressive role are colored green, while the oncogenic ncRNAs are colored red.

Depending on their role in the development of UM, miRNAs can be classified as tumor suppressor miRNAs or oncogenic miRNAs. Oncogenic miRNAs downregulate the transcription of tumor suppressor genes; conversely, downregulation of tumor suppressor miRNAs results in UM progression by allowing their target oncogene to be subsequently upregulated. The MAPK/ERK pathway and PI3K/AKT pathway with its natural inhibitor PTEN have been described as direct targets of many miRNAs in the development of UM [13]. The relationships established between these types are shown schematically in Figure 2. The complete list of miRNAs that have suppressive and oncogenic actions in UM is shown in Table 1; Table 2, respectively, along with information regarding their expression profiles, function, and direct genetic targets. A summary indicating the main characteristics of the UM cell lines mentioned in these tables are shown in Table S1 [14,15,16,17,18,19]. 

#### 2.1.1. miRNA Tumor Suppressors

In UM, most identified miRNAs play a role in inhibiting tumors and are defined by their characteristic of downregulating oncogenes. Yan et al. identified miR-34a as a transcriptional target of p53, thus implying an antitumor effect. UM cell cultures transfected with miR-34a had decreased cell growth and migration. Moreover, miR-34a suppressed the expression of c-Met in addition to decreasing the levels of phosphorylated Akt and other cell cycle-related proteins (Rb, cdc2, and E2F3) [23]. The c-Met tyrosine kinase receptor is implicated in the upstream pathways of PI3K/AKT and Ras/MAPK; it is overexpressed in 60–86% of solid tumors and associated with tumor aggressiveness in UM [30,45]. In addition, the suppressive role of miR-34a was reinforced when LGR4 (leucine-replication-rich G-protein coupled receptor 4) was identified as another of its targets. This receptor has been implicated in processes of embryological development, cell mobility, and metastasis. LGR4 is overexpressed in UM cells, and transfection of miR-34a has been reported to decrease its expression [24]. LGR4 can inhibit the expression of MMP2, which has been shown to be responsible for the migration and invasion of many kinds of tumors, including UM [24]. In parallel, two other miRNAs from the same miR-34 family (miR-34b and miR-34c) have also been described as tumor suppressors in UM. Their levels were found to be decreased in UM cell lines and primary samples [25]. Therefore, expression of miR-34b/c results in reduced cell growth and migration by stopping the cell cycle at G1 [25]. Similar to miR-34a, C-Met has been identified as a target of miR-34b/c; other agents involved in the c-Met signaling pathway, such as p-Akt, CDK4, and CDK6, were also detected as underexpressed upon introduction of miR-34b/c in UM [25]. In a similar way, overexpression of miR-122 and miR-144 can also reduce the expression of c-Met and ADAM10 proteins, resulting in reduced proliferation, migration, and cell cycle progression in UM [26].

Other authors have described miR-137 in UM, finding that its expression was lower in tumoral cells than in normal melanocytes [28]. Transfection of miR-137 into melanocytes induced cell cycle arrest in G1, leading to decreased cell growth. miR-137 transfection also led to decreased levels of MITF, c-Met, and other cell cycle-related proteins such as CDK2 and CDK6 [28]. Similar to the function of miR-137, miR-182 can suppresses the expression of *MITF* and cell cycle-related genes [32]. In another study, it was observed that miR-137 could downregulate the expression of steroid receptor coactivators (SRCs), thus decreasing the tumor’s proliferation and ability to metastasize [46].

Interleukin 10 (IL-10) suppresses the inflammatory response and immune reactions against a variety of tumors [47]. Transfection of miR-15a, miR-185, and miR-211 independently inhibited cell proliferation through specific targeting of the *IL-10Rα* receptor gene [35]. Accordingly, the individual or combined expression of these three miRNAs led to a significant reduction in UM cell proliferation [35].

On the other hand, Liu and coauthors revealed the previously unknown role of miR-9 in UM [20]. Its levels were reduced in highly invasive UM, and evidence suggests that suppression of cell migration and invasion of UM cells in culture occurred through modulation of the expression of NF-KB1, which is involved in proliferation, apoptosis, angiogenesis, and tumor metastasis. Other proteins related to the NF-KB1 signaling pathway are also underexpressed, such as VEGFA, MMP-2, and MMP-9. These proteins participate in a signaling pathway that leads to tumor initiation and progression through the action of MMP (matrix metallopeptidase), which degrades basement membranes and the extracellular matrix, facilitating the spread of tumor cells. This inhibitory role on MMP2/MMP-9 in UM has also been associated with miR-296-3p [48]. Likewise, miR-23a decreases the cell migration capacity by increasing E-cadherin and reversing the epithelial–mesenchymal transition (EMT) process of UM cells through zinc finger protein Zeb1 [22].

The same tumor suppressor role was also found for miR-124a, which targets CDK4, CDK6, Cyclin D2, and EZH2 proteins, with its expression being diminished in UM cells [27]. Interestingly, it was described that treatment with hypomethylating factors successfully restores miR-124a expression, suggesting that this miRNA may undergo reversible epigenetic regulation [27]. Analogously, Liu et al. [33] demonstrated the tumor-suppressive action of miR-216a-5p in UM cell lines by inhibiting HK2 (hexokinase 2), an aerobic glycolysis-limiting enzyme that is overexpressed in numerous human cancers.

It has recently been described that miRNA-145 and miRNA-205 can reduce the proliferation, migration, and invasion of UM cells by targeting the mRNA of their upstream protein NRP1 to regulate the level of expression of CDC42 [31]. CDC42 are small GTPases that have been linked to multiple human cancers and are implicated in the epithelial to mesenchymal transition, cell cycle progression, migration/invasion, tumor growth, angiogenesis, and oncogenic transformation [49]. Other miRNAs that have been described as tumor suppressors in UM capable of decreasing levels of cell growth and invasion are miR-142-3p [29] and miR-224-5p [34]. A complete list of UM-related suppressive miRNAs is summarized in Table 1.

#### 2.1.2. Oncogenic miRNAs

miRNAs that promote the initiation and progression of UM are considered oncogenic miRNAs. Notably, miR-21—as one of the most studied and described oncogenic miRNA—also appears to be overexpressed in other types of tumors [50]. In UM, it causes suppression of *p53* gene expression and increases the expression of the cell-adhesion protein LASP1 and GST-pi protein—an enzyme involved in cell detoxification—protecting tumor cells from cytotoxic drugs. Zhang et al. analyzed the action of the miR-181 members family (miR-181a, miR-181b, miR-181c), finding that their expression was increased in UM cell lines [40]. In particular, miR-181b was reported to inhibit the expression of the tumor-suppressing phosphatase enzyme (CTDSPL) that dephosphorylates tumor suppressor Rb; thus, this miRNA inhibits the role of Rb in increasing cell proliferation [40].

Cheng et al. identified miR-222 as a mediator of the oncological effect of HMBA1 (high-mobility Group A1), a transcription factor already involved in the progression of various tumors [51,52]. The introduction of miR-222 into UM cell lines resulted in an increase in PI3K, p-Akt, and MMP9 [34]. These proteins appear to participate in a signaling pathway that leads to tumor initiation and progression through the action of MMP-9 (matrix metallopeptidase 9), which degrades basement membranes and the extracellular matrix, facilitating the spread of tumor cells [41].

PTEN is a tumor suppressor involved in the PI3K/AKT pathway; its loss of expression is associated with a more aggressive UM, which augurs for future metastasis [53]. PTEN has been identified as a target of miR-367 or miR-454; its levels decrease as they increase. Inhibition of these miRNAs resulted in suppressed cell proliferation, cell cycle progression, and cell migration, while their introduction had the opposite effects, presumably via inhibition of PTEN [42,43]. Furthermore, miR-155 introduction into cultures promoted cell growth and invasion while its inhibition produced contrary effects. Protein I related to the Nedd4 family (NDF1P1) was identified as the target of miR-155. This protein is involved in the ubiquitination and nuclear translocation of PTEN [39].

On the other hand, miR-652 directly represses the expression of HOXA9 and leads to the activation of the HIF-1 signaling pathway promoting tumor proliferation and migration in UM through HK2, a mechanism that was mentioned in the previous section [44]. High expression of miR-92a-3p appears to be associated with proliferation in other types of non-UM cancers [38,54]. Treatment with the histone deacetylase inhibitor MS-275 may reduce the expression of miR-92a-3p and increase the levels of MYC 2-binding protein (MYCBP2), which is a target gene for miR-92a-3p. This would eventually lead to apoptosis of the UM cells [38]. Finally, the transfection of miR-20a into UM cell lines contributed to increased cell invasion and migration, although, in this case, its cellular target has not yet been elucidated [36].

### 2.2. miRNAs in Clinical Applications

#### 2.2.1. miRNAs as Potential Biomarkers for UM

It has been shown that the miRNA expression profiles can be used to distinguish cancerous from normal samples and even allow the classification into different subtypes and clinical stages [55,56]. miRNAs have been found in multiple fluids that allow easy accessibility as well as presenting a high degree of stability because miRNAs are usually released from cells through vesicles and cannot be easily degraded by RNases, allowing them to have long half-lives of up to 24 h [57,58]. These characteristics make miRNAs excellent potential biomarkers that could be combined with other possible serum biomarkers of a protein nature [59,60].

##### Studies on Tissue Samples/Cell Lines

Worley et al. were the first to determine the expression of miRNAs in 24 primary frozen UM tissues through microarray miRNA analysis, correlating the results with prognosis through determining the risk of metastasis based on chromosome 3 abnormalities [61]. They further established that let-7b and miR-199a were the miRNAs that demonstrated the greatest discriminatory potential between Class 1 and Class 2 UM, respectively corresponding to the lowest and highest metastatic risk. Moreover, they describe that through the use of a combination of the six main miRNAs with discriminatory potential (let-7b, miR-143, miR-199a, miR-199*, miR-193b, and miR-652), 100% sensitivity and specificity was achieved in distinguishing Class 1 from Class 2 tumors [61]. Similarly, in another study, five miRNAs (miR-214, miR-146b, miR-143, miR-199a, and miR-134) were found to be differentially expressed in monosomy 3 and disomy 3 UM bearing tumors [62]. The miRNAs that were found to better correlate with development of liver metastases were miR-149 and miR-134 [62]. An extensive TCGA (The Cancer Genome Atlas) UM study analyzing 80 primary UM samples was conducted by Robertson et al., in which they identified four clusters of miRNAs that were associated with chromosome 3 status, risk of metastasis, and DNA methylation profile [63]. Consistent with Worley et al., miR-199a-3p/5p, miR-199b-3p, and let-7b-5p were more highly expressed in UM characterized by chromosome 3 monosomy [61].

In another study, Smit et al. classified 26 UM tissue samples into three groups based on previously described genetic mutations linked to oncological progression (*GNAQ, GNA11, EIF1AX, SF3B1, BAP1*, chromosome 3 monosomy). They identified 13 miRNAs whose expression varied in a differential manner between UM and were linked to its classification as high risk, intermediate, or low risk of metastasis. Five different miRNAs were overexpressed in the high-risk group (miR-132-5p, miR-151a-3p, miR-17-5p, miR-16-5p, and miR-21-5p) and eight were underexpressed (miR-181b-5p, miR-101-3p, miR-378, miR-181a-2-3p, miR-99a-5p, let-7c-5p, miR-1537-3p, and miR-99a-3p). The 106 genes possibly regulated by these miRNAs were also found to be involved in cell cycle regulation pathways and EGF and EIF2 signaling pathways [64].

Wróblewska et al. validated 6 miRNAs (of the 15 previously selected by TCGA) in 46 UM tissue samples for the distinction between primary and metastatic UM. Results corresponded to either elevated (miR-592, miR-346, and miR-1247) or decreased (miR-506 and miR-513c) expression in the metastatic stage. miR-196b did not show quantitative differences in its expression, but was correlated with the expression of BAP1 [65]. In histological samples, Radhakrishnan and coworkers related chromosomal alterations of pairs 1, 3, and 8 with miRNA expression according to whether the samples belonged to tumors that had developed liver metastases or not [66]. They also characterized two different miRNA expression profiles in tumors with or without liver metastasis, finding that a total of 30 miRNAs were differentially expressed [66].

In recent years, different computational techniques have been used to analyze miRNA expression data in the TCGA UM database to identify miRNAs as potential prognostic biomarkers. Falzone et al. determined the prognostic importance of deregulated miRNA based on TCGA data. They determined seven miRNAs associated with tumor stage, vital status, and overall survival, including six that were downregulated (miR-514a-3p, miR-508-3p, miR-509-3-5p, miR-513c-5p, miR-513a-5p, and miR-211-5p) and another six that were upregulated (let-7b-5p, let-7b-3p, miR-224, miR-452, miR-592, and miR-199a-5p) [67]. Additionally, also using bioinformatic analysis, Xin et al. established two groups of UMs according to their prognosis risk based on miRNA analysis. In their study, they inferred that this classification can be established by testing the expression of nine miRNAs (miR-195, miR-224, miR-365a, miR-365b, miR-452, miR-4709, miR-7702, miR-513c, and miR-873), which exert action on target genes related to relevant carcinogenic processes [68]. Finally, in contrast to the above, Larsen and others in their study of 26 UM samples found no association between miRNA expression and the ability to predict metastasis and survival in UM [69]. Table 3 shows the complete list of potential biomarkers determined from studies of miRNA.

##### miRNAs in Serum or Plasma

Stark and coworkers proposed a panel of 17 miRNAs to differentiate UM from other benign melanocytic lesions such as nevi. Thus, by means of PCR, they analyzed the blood of 10 patients with nevus, 50 with localized UM and 5 with metastatic UM. Six of the 17 studied miRNAs showed significant differences between patients with nevus and UM, namely miR-16, miR-145, miR-146a, miR-204, miR-211, and miR-363-3p. Interestingly, the proposed panel of six miRNAs identified UM with 93% sensitivity and 100% specificity when at least four of the miRNAs were used [70].

Russo et al. studied the expression of miRNAs in serum in 14 patients with UM [71]. They found significant differences compared to healthy controls in eight miRNAs. Two miRNAs were overexpressed (miR-146a and miR-523) and six were underexpressed (miR-19a, miR-30d, miR-127, miR-451, miR-518f, and miR-1274b) in UM. After validation, only miR-146a showed significant differences in upregulation between histological UM samples [71]. On the other hand, Achberger et al. studied the blood of six patients diagnosed with UM and identified miRNAs with immune regulation capacity. The plasma levels of miR-20a, miR-125b, miR-146a, miR-155, miR-181a, and miR-223 were found to be increased in patients with UM as compared to healthy controls. Of these, levels of miR-20a, miR-125b, miR-146a, miR-155, and miR-223 were found to be increased in metastatic stages, while miR-181 was decreased [72].

A study by Ragusa et al. attempted to characterize the miRNA expression profiles in vitreous humor, vitreous humor exosomes, and serum of six patients with UM as compared to six healthy subjects as controls [73]. Ninety percent of the miRNAs were shared between the miRNA profiles of vitreous humor and vitreous exosomes. This suggests the possibility that alterations in vitreous humor exosomes are responsible for those found in the vitreous humor miRNA profile. miR-618 was underexpressed in vitreous humor but overexpressed in vitreous exosomes and serum, showing the unique characteristics of the miRNA profile in vitreous humor. The levels of miR-21, miR-34a, and miR-146a were increased in vitreous humor, vitreous exosomes, and histological samples. Interestingly, miR-146a was upregulated in the serum of UM patients, as well as in serum exosomes [73].

Finally, Triozzi et al. monitored antiangiogenic treatments in UM by detecting miRNAs in the blood. They reported the plasma levels of these miRNAs and those of miR-216 and miR-16 were shown to change following treatment with interferon-alfa-2b but not dacarbazine [74]. In a later report, they detected overexpression of miR-92b, miR-223, and miR-199-5p in plasma in samples of UM with chromosome 3 monosomy [74].

A summary of the different studies is presented in Table 4 in a schematic way. It can be seen how the miR-146a is reflected in several studies with increased values in body fluids of patients with primary and metastatic UM. Although uveal melanoma is barely studied, miR-146a has been described to increase proliferation of skin melanoma cells in culture and able to form tumors in mice [75]. In addition, miR-146a is a MITF target that is responsible for regulating the development and survival of melanocytes and may also be involved in the pigmentation and proliferation processes [76]. This may make miR-146a a serious candidate as serum biomarker in UM, although further studies would be needed to statistically validate its diagnostic power in larger cohorts of patients.

#### 2.2.2. miRNAs as Immune Regulators

miRNAs are becoming increasingly recognized in the regulation of immune responses. Natural killer cells (NK cells) inhibit oncological progress in UM by mediating cellular cytolysis and apoptosis of cancer cells [77,78]. There is evidence that cancer stem cells (CSC) are a key factor in UM metastasis [79]. Joshi et al. reported that UM CSCs could synthesize miRNA regulators of NK cells, including miR-181a, miR-146a, miR-20a, miR-223, and miR-155. The transfer of antimiR-155 to MUM2B cells (a highly invasive metastatic UM cell line) increased the sensitivity of NK cells. In addition, high levels of miR-155 expression and secretion are detected in CSC UMs, which suggests that suppression of NK cells by miR-155 contributes to metastatic progression [80]. On the other hand, miR-199a-5p participates in regulatory T-cell regulation [81] and miR-223 regulates myeloid-derived suppressor cells (MDSCs) [82].

## 3. Long Non-Coding RNAs in UM

Long non-coding RNAs (lncRNAs) are a type of RNA with a length exceeding 200 nucleotides and without protein coding potential [5,83,84,85]. Modifications of the epigenetic sphere include the regulation of imprinting, conformational changes at the chromosomal level and chromatin remodeling, modifications in the methylation of histone proteins and DNA, and regulation through ncRNAs [86,87].

It is well known that lncRNAs are capable of carrying out their functions through two mechanisms, either by acting directly on neighboring genes generally found on the same chromosome or by regulating genes at a distance, including those on other chromosomes [86,88]. In addition, it has been shown that lncRNAs can interact with microRNAs [12] and may even act as specific competing endogenous RNAs (ceRNAs) to suppress the function of the miRNAs themselves [89].

Several studies have shown that lncRNAs are able to perform their functions through interaction with the Enhancer of zeste homolog 2 (EZH2), a member of the polycomb family (PcG) capable of regulating the cell cycle through interaction with transcriptional factors, chromatin remodeling, and modification of nucleosomes [90,91,92]. Numerous studies have previously shown that upregulation of EZH2 is correlated with cell proliferation and poor tumor prognosis [93]; thus, in UM, a high level of EZH2 expression has been demonstrated, suggesting that it is an oncogenic protein in this type of neoplasm [90,94,95]. However, despite this carcinogenic effect, other studies have suggested that EZH2 may also have an antitumor effect in some neoplasms since its inhibition favors tumor progression [90].

### 3.1. Plasmacytoma Variant Translocation 1 (PVT1)

The levels of lncRNA PVT1 (Chr 8q24.21) were found to be considerably higher in UM compared to normal tissues [21,90]. It has an oncogenic function in the initiation and development of metastasis of various neoplasms, including UM [96,97,98,99]. The silencing of PVT1 impedes the biological functions of UM cells, resulting in the inhibition of proliferation, S-phase sequestration, and migration in addition to inducing apoptosis [21].

The mechanisms by which PVT1 exerts its carcinogenic function in UM are through the positive regulation of EZH2 expression [90] as well as its function in regulating the miR-17-3p/MDM2/p53 axis. Wu et al. [21] showed that there is specific binding between lncRNA PVT1 and miR-17-3p, which implies inhibition of the expression of this microRNA. In addition, they demonstrated low expression of miR-17-3p and a high level of MDM2 in UM tissues, with a targeting relationship between both. Thus, the miR-17-3p is able to inhibit the expression of MDM2, ultimately facilitating the transcriptional activation of the tumor suppressor protein p53 and reduction in the tumorigenic capacity. In conclusion, lncRNA PVT1 favors tumor emergence and development by binding to and inhibiting the expression of miR-17-3p, promoting the expression of MDM2 and the inactivation of p53.

Xu et al. demonstrated the association between a high expression of PVT1 and a number of clinicopathological parameters of poor prognosis in UM, such as advanced age, histological epithelioid-type, presence of extra-scleral extension, and distant metastasis [96]. However, it was not correlated with the thickness or size of the tumor mass. Overall, PVT1 overexpression was correlated with a higher mortality rate and was able to independently predict poor overall survival of such patients with UM (Figure 2).

### 3.2. CASC15-New-Transcript 1

Nuclear lncRNA CASC15-new-transcript 1 (CANT1 or CASC15-NT1), present in human uveal tissue, is an isoform of non-coding lncRNA CASC15 (Chr 6p22.3). Both CASC15 and CANT1 are downregulated in UM while an inverse expression patten is observed in normal cells [100].

CANT1 acts as a tumor suppressor, inhibiting both cell migration and tumorigenesis of UM, for which it is able to trigger and modulate a non-coding cascade (CANT1–JPX/FTX–XIST) [100]. As with CANT1, these three potential regulatory targets—*JPX, FTX*, and *XIST*—have low levels of expression in UM tissues. Inhibition of JPX/FTX transmits the signal to XIST, decreasing its expression levels and completing the sequence that ultimately leads to both activation of tumor growth and stimulation of metastatic capacity.

### 3.3. Metastasis-Associated Lung Adenocarcinoma Transcript 1 (MALAT1)

MALAT1 (Chr 11q13.1) has been reported to be able to function as a ceRNA since it regulates the expression of miRNAs such as miR-34 or miR-183 in cutaneous melanoma [101,102]. As with other types of tumors, MALAT1 acts as an oncoRNA in UM cells, and its abundance is upregulated [103,104]. Two main axes have been described by which lncRNA MALAT1 performs its oncogenic function:

(a) Sun et al. demonstrated that MALAT1 inhibits miR-140 and, thus, promotes the protein expression of its target genes, *Slug* and *ADAM10*, in order to induce proliferation, migration, colony formation, and invasion of UM cell lines [103]. (b) Wu et al. deciphered the MALAT1/miR-608/HOXC4 axis in UM, showing that MALAT1 is capable of enhancing HOXC4 expression through the molecular interaction and inhibition of miR-608, ultimately promoting in vitro UM cell proliferation, invasion, and migration and in vivo tumor growth [104] (Figure 2).

### 3.4. Homeobox A11 Antisense (HOXA11-AS)

Lu et al. described a high level of expression of HOXA11-AS (Chr 7p15.2) in UM tumor samples. This is an oncogenic lncRNA, which promotes tumorigenesis by inducing cell proliferation and invasion while inhibiting apoptotic events [94]. The pathways by which HOXA11-AS performs its functions are the following: (a) through direct interaction with *EZH2*, to silence the transcription of the cell cycle regulation gene *p21*; (b) binding directly to miR-124 and inhibiting its expression; and (c) acting as a ceRNA of miR-124, since degradation of HOXA11-AS enzyme causes a decrease in EZH2 levels (Figure 2).

### 3.5. Small Nucleolar RNA Host Gene 7 (SNHG7)

The expression of SNHG7 (Chr 9q34.3) is reduced in UM tissues, and especially in cases where disease is already metastatic [95]. Thus, low levels of SNHG7 expression are associated with worse prognosis in UM as well as a higher proportion of epithelioid cells, poor clinical staging, lower tumor-free survival, and a higher mortality rate. SNHG7 is inversely associated with EZH2, and high levels of EZH2 expression were found to be correlated with a poor histological type and higher clinical TNM stage [95] (Figure 2).

### 3.6. Ferrtin Heavy-Chain 1 Pseudogene 3 (FTH1P3)

It was found that FTH1P3 (Chr 2p23.3) is upregulated in UM tissues, and that such elevated levels of expression favor proliferation and migration while promoting cell cycle progression. The lncRNA FTH1P3 therefore functions as an oncoRNA that induces UM progression through target interaction inversely correlated with microRNA-224-5p (miR-224-5p) levels [105]. Ultimately, the increase in FTH1P3 levels implies secondary elevation of the protein expression of Rac1 and Fizzled 5 as they are direct target genes of miR-224-5p [97] (Figure 2).

### 3.7. Purinergic Receiver P2X7 Variant3 (P2RX7-V3)

P2RX7-V3 (Chr 12q24.31) is specifically expressed in UM and functions as an oncogene, present at high levels and promoting proliferation, migratory capacity, cell invasion, tumor growth, and metastasis [106]. Furthermore, by silencing P2RX7-V3, it was demonstrated that a wide variety of genes are deregulated and that the main target signaling pathway by which it performs its carcinogenic functions is the PI3K/AKT pathway [106].

### 3.8. Rhophilin RHO Gtpase Binding Protein 1 Antisense 1 (RHPN1-AS1)

Lu et al. demonstrated that RHPN1-AS1 (Chr 8q24.3) is an oncoRNA as it is overexpressed in UM cell lines and induces the proliferation, migration, invasion, and growth of tumor mass by deregulation of various biologic functions and pathways [107]. Their study was carried out by means of gene expression analysis after knockdown of RHPN1-AS1, observing that the most affected biological processes are angiogenesis, cell adhesion, and organization of the extracellular matrix, and that the most regulated signaling pathways are, among others, the metabolism of nicotinate and nicotinamide as well as the transforming growth factor-beta (TGF-β) pathway, whose constituent genes are stimulated, suggesting that RHPN1-AS1 favors UM progression by participating in this signaling pathway.

### 3.9. Pax6 Upstream Antisense RNA (PAUPAR)

At the cellular level, PAUPAR (Chr 11p13) is mainly located in the nucleus and is considered a neoplastic suppressor that inhibits tumor formation, both in vitro and in vivo, in addition to suppressing metastatic progression and dissemination in UM [108]. PAUPAR induces silencing of HES1 [108], a mediator of the Notch signaling pathway which plays a key role in the survival of immature melanoblasts and melanocytic stem cells by inhibiting the onset of apoptotic phenomena [109]. PAUPAR expression is reduced in UM cell lines and tissues while HES1 is highly expressed compared to normal healthy tissues. Similarly, the Notch pathway is activated [110], resulting in increased migration capacity and cell colony formation [108].

### 3.10. Other lncRNAs

lncRNA-numb (Chr 14q24.3) acts as a tumor suppressor, and its low levels of expression may be associated with the progression of UM by favoring cell proliferation and invasion [111].

On the other hand, recently, the high expression of the lncRNA SNHG15 (Chr 7p13) has been associated to clinical-pathological variables of UM related to the bad prognosis, including age, diameter of the tumor, pathological type, and extra scleral extension. At present, the impact of SNHG15 in UM is still not clear. Therefore, it is necessary to elucidate the biological mechanism of the SNHG15 in UM [112].

## 4. Conclusions

In this review, we have attempted to establish a schematic approach to the new topic of ncRNAs in UM. In the last decade, these small molecules have become very important in cancer research: in particular, the number of published works related to UM has multiplied exponentially. The number of ncRNAs being newly described new each year makes it impossible to compile all the available information in a simple way. It seems clear that ncRNAs will play an important role in the diagnosis, prognosis, and treatment of UM in the near future.

ncRNAs along with their regular target genes participate in multiple biological processes in tumors that makes them essential for the proliferation and continued metastasis of tumors. This makes them potential targets and regulators of the therapeutic response in UM. miRNA let-7b appears to suppress the expression of cyclin D1 in UM cells and enhance the radiosensitivity of UM through cell cycle arrest. This characteristic of let-7b could allow it to act as a radiosensitivity enhancer and improve the therapeutic effects of local radiation in the treatment of UM [113]. Anti-PD-1/PD-L1 antibodies (such as pembrolizumab, nivolumab) are used as drugs in the treatment of metastatic UM. PD-L1 is constitutively expressed in tumor cells and interacts with PD-1 to deactivate T cells, leading to immune escape [114]. Audrito et al. found an inverse correlation between miR-17-5p and the expression of PD-L1 in uveal melanoma cell lines [115]. A decrease in the expression of PD-1 was found in mice with miR-28-transferred melanoma, meaning that the miR-28 could silence PD-1 and reverse the exhaustive state of T cells [116].

In an interesting article, Sharma et al. described how BAP1 mutations can change the expression of a tumor-specific miRNA network, resulting in alterations to UM behavior [117]. BAP1 is, conceptually, a driver gene in UM and its alteration has been linked to an increased risk of metastasis. All this reinforces the concept that ncRNAs could serve as excellent prognostic markers, with the added bonus that they can be found in multiple body fluids along with the advantage that they can be obtained noninvasively. Unfortunately, at present, multiple studies have produced very few miRNAs that appear to be common biomarkers—in some cases, even producing conflicting results. Reasons for this could be the use of small samples and heterogeneity in the inclusion processes or in the quantification methods [118].

Though a wide variety of ncRNAs have been studied that are correlated with the pathogenesis of UM, their function and expression remain unknown in many cases. Research into the different types of ncRNAs as well as their underlying mechanisms of action may have vitally important diagnostic, therapeutic, and prognostic implications in UM. This is further complicated by the recent discovery of miRNA isoforms (isomiRs) that were initially ruled out as aberrant sequence artifacts. Some miRNA arms can produce more than 30 different isomiRs, and each isomiR of the same miRNA can target different mRNAs. Therefore, isomiR expression could greatly increase the number of miRNA-regulated target mRNAs [119,120]. Londin et al. found examples of previously reported miRNA loci that produce abundant isomiRs in UM and that their expression was correlated with clinical attributes [121].

To conclude, we hope that future studies will allow us to have a better understanding of the participation of ncRNAs in UM. We believe that these studies should include a greater number of samples and adopt bioinformatic tools for predictive analyses to generate preliminary findings that will require subsequent clinical validation.

## Figures and Tables

**Table 1:** miRNAs with UM tumor suppressor action. A list of known miRNAs with tumor suppression role in UM is shown indicating their chromosomal location, cell/tissue where it was described, their inhibitory action, gene target, existence of experimental validation, and reference. A summary indicating the main characteristics of the UM cell lines mentioned in this table are shown in Table S1 [14,15,16,17,18,19].

miRNA	Location ^†^	Sample Type	Inhibitory Events	Validation	Target	Reference
miR-9	1q22	Highly invasive cell lines: MUM2B and C918	Cell migration and invasion	MA, IA, PA, RT-PCR, WB, FRA	*NF-kB1*	[20]
miR-17-3p	13q31.3	UM patient tumor tissues; UM cell lines: OCM-1A, MUM-2C, C918, and MUM-2B	Cell proliferation and metastasis	RT-PCR, PA, MA, IA, Dual LUCA, WB, ATM	*MDM2*	[21]
miR-23a	19p13.12	UM cell lines: OCM-1; UM patient tumor tissues	Cell migration	RT-PCR, MA IF, WB	*Zeb1*	[22]
miR-34a	1p36.22	UM cell lines: M17, M21, M23, and SP6.5; UM patient tumor tissues	Cell proliferation and migration	PA, MA, IA, Dual LUCA, IF, WB	*C-MET* *LGR4*	[23,24]
miR-34b/c	11q23.1	UM cell lines: SP6.5; UM patient tumor tissues (upregulated in doxorubicin- and epigenetic drug-treated SP6.5 cells)	Cell proliferation and migration	PA, AA, MA, LUCA, WB	*C-MET*	[25]
miR-122	18q21.31	UM cell lines: 92.1, MEL270, OMM2.5, UPMM2, and UPMM3; TCGA UM dataset, UM patient tumor tissues	Cell proliferation and migration	RT-PCR, LUCA, WB	*C-MET, ADAM10*	[26]
miR-124a	3 loci: 8p23.1 8q12.3 20q13.33	UM cell lines: M17, M21, M23, and SP6.5; UM patient tumor tissues	Cell proliferation, migration, and invasion	PA, MA, IA, RT-PCR, ATM, WB	*CDK4, CDK6*, *cyclin D2, EZH2*	[27]
miR-137	1p21.3	UM cell lines: OMM1.3, Mel202, 92.1, OMM1, OCM1, and OCM3	Cell proliferation	RT-PCR, PA, LUCA, WB	*MITF* *CDK6*	[28]
miR-142-3p	17q22	UM cell lines: M17 and SP6.5; UM patient tumor tissues	Cell proliferation, migration, and invasion	RT-PCR, PA, MA, IA, AA, LUCA, WB	*CDC25C, TGFβR1, GNAQ, WASL, RAC1*	[29]
miR-144	17q11.2	UM cell lines: MUM-2B, C918, MUM-2C, and OCM-1A; UM patient tumor tissues	Cell proliferation and migration	LUCA, WB, PA, IA	*C-MET*	[30]
miR-145	5q32	UM cell lines: MUM-2B and OCM-1; UM patient tumor tissues	Cell proliferation and promotes apoptosis	RT-PCR, PA, IA, Dual LUCA, WB	*IRS-1* *NPR1*	[31]
miR-182	7q32.2	UM cell lines: M23 and SP6.5; UM patient tumor tissues;(upregulated by p53 activation)	Cell proliferation, migration, and invasion	RT-PCR, PA, MA, LUCA, ATM, WB	*MITF, BCL2, cyclin D2*	[32]
miR-205	1q32.2	UM patient tumor tissues (both high- and low invasive UM)	Cell proliferation and promotes apoptosis	PA, IA, Dual LUCA, WB	*NPR1*	[31]
miR-216a-5p	2p16.1	UM cell line: MUM-2B; UM patient tumor tissues	Cell proliferation	PA, RT-PCR, LUCA, GA, WB, ATM, IHC	*HK2*	[33]
miR-224-5p	Xq28	UM cell line: OCM-1A; UM patient tumor tissues	Cell proliferation, migration, and invasion	RT-PCR, PA, MA, IA, LUCA, WB	*PIK3R3/AKT3*, *FTH1P3, RAC1, Fizzled 5*	[34]
miR-15a, miR-185, miR-211	13q14.2 22q11.21 15q13.3	Cell line: OCM-1; UM patient tumor tissues	Cell proliferation	RT-PCR, PA, WB	*IL-10Rα*	[35]

Abbreviations: RT-PCR: real time polymerase chain reaction; PA: proliferation in vitro assay; MA: migration in vitro assay; IA: invasion in vitro assay, AA: apoptosis assay; WB: western blot immunodetection; LUCA: luciferase expression assays; IF: Immunofluorescence; IHC: Immunohistochemistry; ATM: animal tumor models, GA: glycolysis assay. ^†^ Information obtained from GeneCards database (www.genecards.org).

**Table 2:** miRNAs with oncogenic action. A description of known miRNAs with oncogenic role in UM is shown indicating their chromosomal location, cell/tissue where it was described, their tumor promoting effect, gene target, existence of experimental validation, and reference. A summary indicating the main characteristics of the UM cell lines mentioned in this table are shown in Table S1 [14,15,16,17,18,19].

miRNA	Location ^†^	Sample Type	Pro-Tumorigenic Events	Validation	Target	Reference
miR-20a	13q31.3	MUM-2B and MUM-2C metastatic UM cells; UM patient tumor tissues	Cell proliferation, migration, and invasion	PA, IA, MA, RT-PCR	Not validated	[36]
miR-21	17q23.1	OCM-1, M619 and MUM-2B UM cells	Cell proliferation, migration, and invasion	PA, AA, MA, Dual LUCA, WB	*p53*	[37]
miR-92a-3p	2 loci: 13q31.3 Xq26.2	OCM-1 UM cells	Inhibition of apoptosis	LUCA, WB	*MYCBP2*	[38]
miR-155	21q21.3	OCM-1A, MUM-2C, C918, and MUM-2B UM cells; UM patient tumor tissues	Cell proliferation and invasion	PA, IA, LUCA, WB	*NDFIP1*	[39]
miR-181 (family)	Chr 1, 9, and 19	SP6.5, VUP, OCM1, MUM2b, and 92-1; UM patient tumor tissues	Cell proliferation	Dual LUCA, WB, RT-PCR	*CTDSPL*	[40]
miR-222	Xp11.3	C918 and MUM-2B UM cells	Cell proliferation and migration	AA, PA, MA, WB	*PI3K/Akt/MMP-9*	[41]
miR-367	4q25	UM cell lines: M17, M23, MUM-2B, and C918; UM patient tumor tissues	Cell proliferation and migration	LUCA, PA, MA, WB	*PTEN*	[42]
miR-454	17q22	UM cell lines: OCM-1A, MUM-2B, MUM-2C, and C918; UM patients tumor tissues	Cell proliferation, colony formation, and invasion	RT-PCR, PA, IA, LUCA, WB	*PTEN*	[43]
miR-652	Xq23	MUM-2B and MEL270 UM cell lines; UM patient tumor tissues	Cell proliferation and migration	PA, MA, Dual LUCA, RT-PCR, WB	*HOXA9*	[44]

Abbreviations: RT-PCR: real time polymerase chain reaction; MA: in vitro cell migration assay; IA: in vitro invasion assay; PA: proliferation in vitro assay; WB: western blot immunodetection; LUCA: luciferase expression assay; AA: apoptosis assay. ^†^ Information obtained from GeneCards database (www.genecards.org).

**Table 3:** miRNAs in association with an increased risk of uveal melanoma metastasis.

Sample Type	Over Expressed		Under Expressed	Reference
	High Risk UM Metastatic	Low Risk UM Metastatic	High Risk UM Metastatic	
24 UM patient frozen tumor tissues	**let-7b**, **miR-143**, miR-193b, **miR-199a**, miR-199 *, miR-652			[61]
60 UM patient tumor tissues and 11 control tissues	miR-33a, miR-99a, miR-135b, miR-196a, miR-325, miR-497, miR-512-5p, miR-549, miR-556-5p, miR-585, miR-640, miR-885-5p ^†^	let-7e, miR-1, miR-10a, miR-18a, miR-19b-1, miR- 26a-2, miR-34c-5p, miR-129, miR-133a, miR-154, miR-181a, miR-218, miR-369-3p, miR-377, miR-376c, miR-493, miR-495, miR-586 ^††^		[66]
26 UM patient tumor tissues	miR-16-5p, miR-17-5p, **miR-21-5p**, miR-132-5p, miR-151a-3p ^‡^		let-7c-5p, miR-99a-3p, miR-99a-5p, miR-101-3p, miR-181a-2-3p, miR-181b-5p, miR-378d ^‡^, miR-1537-3p	[64]
50 UM patient tumor tissues	miR-346, **miR-592**, miR-1247		miR-506 and **miR-513c**	[65]
86 UM patient tumor tissues	miR-134, miR-143, miR-146b, miR-149, miR-199a, miR-214			[62]
TCGA UM database	**Let-7b**, **miR-21**, miR-29b, miR-142, **miR-143**, miR-144, miR-145, miR-150, miR-155, **miR-199a**, **miR-199b**, miR-330, miR-486 ^‡^		miR-30c, miR-181a-5p, miR-335, miR-140, Xq27.3 group	[63]
TCGA UM database	miR-195, miR-224, miR-365a, miR-365b, miR-452, miR-4709, miR-7702		**miR-513c**, miR-873	[68]
TCGA UM database	**let-7b**(5p/3p), **miR-199a-5p**, miR-224-5p, miR-452-5p, **miR-592**		miR-211-5p, miR-508-3p, miR-509–3-5p, miR-513a-5p, **miR-513c-5p**, miR-514a-3p	[67]

The repeated miRNAs appear in bold. ^†^ Genomic Location (Chromosome 3); ^††^ Genomic Location (Chromosome 6); ^‡^ Genomic Location (Chromosome 8). * Information obtained from GeneCards database (www.genecards.org).

**Table 4:** Circulating MicroRNAs as potential biomarkers in UM.

Sample Type	miRNA	Levels	Differentiate (Ratio)	
Vitreous humor and serum (± exosomes)	Vitreous humor (+ exosomes): miR-21, miR-34a, **miR-146a** Serum (+ exosomes): **miR-146a**	↑	Primary/Control	[73]
Serum (14 patients with UM vs. 14 controls)	**miR-146a**	↑	Primary/Control	[71]
	miR-19a, miR-30d ^‡^, miR-127, miR-451, miR-518f, miR-1274b	↓		
Plasma (33 UM M3/22 UM D3/26 controls)	miR-92b, miR-199a-5p, miR-223	↑	M3/D3 M3/Control	[74]
Plasma (6 patients with UM vs. 26 controls)	miR-20a, miR-125b, **miR-146a**, miR-155, miR-181a, miR-223	↑	Primary/Control	[72]
	miR-20a, miR-125b, **miR-146a**, miR-155, miR-223	↑	Metastatic/Primary	
	miR-181a	↓		
Blood (10 uveal nevus, 50 localized UM, 5 metastatic UM)	miR-16, miR-145, **miR-146a**, miR-204, **miR-211**, miR-363-3p	↑	Primary/Metastatic Primary/Nevus	[70]
	**miR-211**	↑	Metastatic/Primary	
	miR-204	↓		

The repeated miRNAs appear in bold. ^‡^ Genomic Location (Chromosome 8). No miRNA in this table is located in Chr 3 or Chr 6. ↑ Overexpressed; ↓ Underexpressed. Information obtained from GeneCards database (www.genecards.org).

## Supplementary Materials

The following are available online at https://www.mdpi.com/2072-6694/12/10/2944/s1, Table S1: Characteristics of the UM cell lines used in the studies in Table 1 and Table 2.
