# Supplementary material for: The Role of Non-Coding RNAs in Uveal Melanoma
Source: Cancers (Basel). 2020 Oct 12;12(10):2944. doi: 10.3390/cancers12102944 (PMC7600503; doi:10.3390/cancers12102944)
Supplement: Supplementary file 1 [file cancers-12-02944-s001.pdf]

The Role of Non-Coding RNAs in Uveal Melanoma

Manuel Bande, Daniel Fernandez-Diaz, Beatriz Fernandez-Marta, Cristina Rodriguez-Vidal, Nerea Lago-Baameiro, Paula Silva-Rodríguez , Laura Paniagua, María José Blanco-Teijeiro, María Pardo and Antonio Piñeiro

Table S1. Characteristics of the UM cell lines used in the studies in Tables 1 and 2.

Cell line	Origin	GNAQ mutations	GNA11 mutations	BAP1 mutations	BRAF mutations	Status of Chr 3	Status of Chr 6	Status of Chr 8	Information
92.1	Primary tumor	Q209L	-	-	-	disomy 3	gain 6p	gain 8q	
MEL202	Primary tumor	Q209L	-		-	disomy 3	gain 6p loss 6q	extra 8 (6 copies)	
OMM2.3	Metastatic (subcutaneous)	Q209P	-	-	-	disomy 3	6p tetrasomy loss 6q	contradictory data (extra 8, gain 8q)	
MEL270	Primary tumor	Q209P	-	-	-	disomy 3, loss 3p24, loss 3q21.2–3q24	6p tetrasomy loss 6q	contradictory data (extra 8, gain 8q)	
OMM2.5	Metastatic (liver)	Q209P	-		-		6p tetrasomy	contradictory data (extra 8, gain 8q)	
UPMM2	Primary tumor	Q209L	-		-	isodisomy			
UPMM3	Primary tumor	Q209P	-		-	monosomy 3			
OCM-1	Primary tumor	-	-	-	V600E	disomy 3	net loss of chr 6	loss of 8p	Poorly invasive potential
OCM-3	Primary tumor	-	-	-	V600E	disomy 3			
SP6.5	Primary tumor	-	-		V600E				
MUM2C	Metastatic (liver)	-	-		V600E				Poorly invasive potential
C918	Primary tumor				-	loss 3p	normal	i(8)(q10)	Highly invasive potential
MUM2B	Metastatic (liver)				-				Highly invasive potential
M17	Primary tumor								Caucasian patients with primary choroid melanoma

M21	Primary tumor							Caucasian patients with primary choroid melanoma
M23	Primary tumor							Caucasian patients with primary choroid melanoma
M619	Primary tumor			-	del(3)(p13)		i(8)(q10)	Highly invasive potential
VUP	Primary tumor							
D78	Human melanocyte cell line							

© 2020 by the authors. Licensee MDPI, Basel, Switzerland. This article is an open access article distributed under the terms and conditions of the Creative Commons Attribution (CC BY) license (http://creativecommons.org/licenses/by/4.0/).
